# Detection of Bovine Antibodies against a Conserved Capsid Epitope as the Basis of a Novel Universal Serological Test for Foot-and-Mouth Disease

**DOI:** 10.1128/JCM.01527-19

**Published:** 2020-05-26

**Authors:** A. S. Asfor, N. Howe, S. Grazioli, S. Berryman, K. Parekh, G. Wilsden, A. Ludi, D. P. King, S. Parida, E. Brocchi, T. J. Tuthill

**Affiliations:** a The Pirbright Institute, Woking, United Kingdom; b Istituto Zooprofilattico Sperimentale della Lombardia e dell’Emilia Romagna, Brescia, Italy; University of Tennessee at Knoxville

**Keywords:** FMDV, conserved capsid epitope, ELISA, diagnosis, conserved capsid, epitope, foot-and-mouth disease virus, serology

## Abstract

Diagnostic tests for foot-and-mouth disease (FMD) include the detection of antibodies against either the viral nonstructural proteins or the capsid. The detection of antibodies against the structural proteins (SP) of the capsid can be used to monitor seroconversion in both infected and vaccinated animals. However, SP tests need to be tailored to the individual FMD virus (FMDV) serotype and their sensitivity may be affected by antigenic variability within each serotype and mismatching between test reagents. As a consequence, FMD reference laboratories are required to maintain multiple type-specific SP assays and reagents. A universal SP test would simplify frontline diagnostics and facilitate large-scale serological surveillance and postvaccination monitoring. In this study, a highly conserved region in the N terminus of FMDV capsid protein VP2 (VP2N) was characterized using a panel of intertype-reactive monoclonal antibodies. This revealed a universal epitope in VP2N which could be used as a peptide antigen to detect FMDV-specific antibodies against all types of the virus. A VP2-peptide enzyme-linked immunosorbent assay (VP2-ELISA) was optimized using experimental and reference antisera from immunized, convalescent, and naïve animals (*n* = 172). The VP2-ELISA is universal and simple and provided sensitive (99%) and specific (93%) detection of antibodies to all FMDV strains used in this study. We anticipate that this SP test could have utility for serosurveillance during virus incursions in FMD-free countries and as an additional screening tool to assess FMD virus circulation in countries where the disease is endemic.

## Abstract (precis)

Diagnostic tests for foot-and-mouth disease (FMD) include the detection of antibodies against either the viral nonstructural proteins or the capsid. The detection of antibodies against the structural proteins (SP) of the capsid can be used to monitor seroconversion in both infected and vaccinated animals. However, SP tests need to be tailored to the individual FMD virus (FMDV) serotype and their sensitivity may be affected by antigenic variability within each serotype and mismatching between test reagents.

## INTRODUCTION

Foot-and-mouth disease (FMD) is an economically devastating viral disease of cloven-hoofed animals with a global distribution. It limits access to markets for developing countries, and outbreaks in otherwise FMD-free countries are expensive to control (as in the United Kingdom in 2001, Japan in 2010, and the Republic of Korea in 2010 and 2011) (1, 2). FMD virus (FMDV) is a single-stranded, positive-sense, RNA virus belonging to the genus *Aphthovirus* in the family *Picornaviridae*. The virus exists as seven serotypes (O, A, C, Asia 1, Southern African Territories 1 [SAT1], SAT2, and SAT3) as well in the form of numerous and constantly evolving strains showing a spectrum of antigenic diversity.

The nonenveloped picornavirus capsid has icosahedral symmetry and a diameter of approximately 30 nm and is composed of 60 copies each of capsid proteins VP1, VP2, VP3, and VP4. VP1, VP2, and VP3 are the major components of the capsid, while VP4 is a small (approximately 12-kDa) internal protein which lies on the inside surface of the capsid around the 5-fold axes of symmetry, where it is thought to stabilize interactions between pentameric capsid subunits (3, 4). During the replication cycle of FMDV, eight different viral nonstructural proteins (NSPs) and additional precursors are generated which are potential serological targets for diagnostic assays (5). The presence of antibodies (Abs) against NSPs can be used to differentiate infected and vaccinated animals (DIVA) because such antibodies are produced only after infection and are not elicited after administration with purified vaccines. In addition, the interserotypic conservation of the NSPs means that this type of test is compatible with all serotypes of FMDV. Hence, NSP tests can be used as generic screening tools to support national programs to ascertain the Office International des Epizooties (OIE) status of FMD freedom with or without vaccination (6–8). However, the specificity of these tests is less than 100% (9), and testing algorithms that are designed to confirm absence of FMDV circulation in large populations usually adopt screening and confirmatory serological assays with covariant rates of false positivity (7–9). In this context, enzyme-linked immunosorbent assays (ELISAs) that measure levels of FMDV-specific antibodies directed at capsid structural proteins (SP) are widely used to augment NSP tests for serosurveillance activities (10–13). One of the international standard tests for FMDV antibody detection is the virus neutralization test (VNT) (14). However, the VNT is laborious, rendering large-scale serological testing difficult. In addition, the procedure requires live virus, thus confining the test to high-containment laboratories in countries where the disease is nonendemic. SP ELISAs with high diagnostic sensitivity are also available for certification of animals as free from FMD prior to import and export for serological confirmation of FMDV infection, for postvaccination monitoring, and for the demonstration of vaccine efficacy (14). However, SP assays need to be tailored to individual serotypes; as a consequence, each FMD Reference Laboratory must maintain parallel assay systems to accommodate the possibility of FMD outbreaks due to different virus serotypes.

A number of monoclonal antibodies (MAbs) with cross-reactivity against multiple FMDV serotypes have previously been reported (15–17). The recognition sites for some of these MAbs have been mapped to a highly conserved region at the N terminus of VP2 (15–17). In this study, the highly conserved N terminus of FMDV capsid protein VP2 (VP2N) was characterized using a panel of cross-reactive MAbs. This revealed a universal epitope in VP2N which has been investigated as a peptide antigen for use in detection of FMDV-specific antibodies in serum samples. We sought to develop a peptide antigen ELISA to detect FMDV-specific antibodies in bovine serum samples from animals infected or vaccinated with any serotype of FMDV.

## MATERIALS AND METHODS

### Cell lines and viruses.

The IBRS-2 (pig kidney) cell line and the BHK-21 (baby hamster kidney 21) cell line, used for FMD virus propagation and immunoassays, were maintained either in Dulbecco’s modified Eagle’s medium or in Dulbecco’s minimum essential medium (DMEM; Thermo-Fisher Scientific, United Kingdom) supplemented with 10% heat-inactivated fetal bovine serum (FBS; Thermo-Fisher Scientific, United Kingdom) and 100 U of penicillin-streptomycin (Sigma) per ml. FMDV strains used are indicated in each relevant paragraph.

### Peptides.

**FIG 1:**
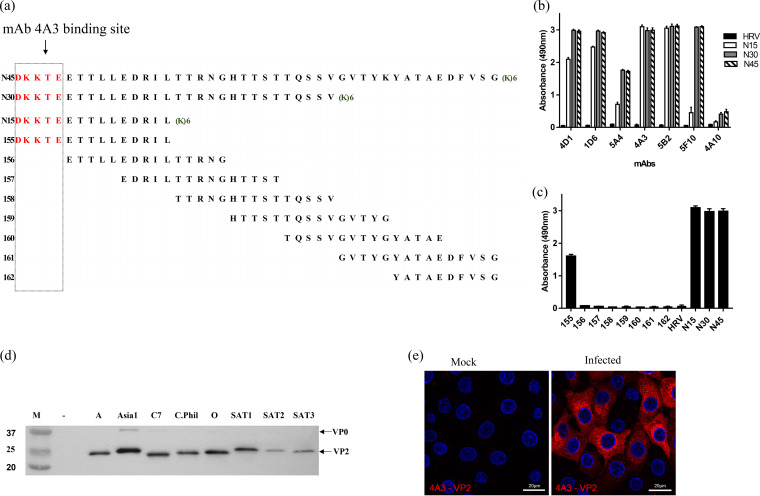
FMDV heterotypic-reactive MAbs recognize the N terminus of VP2. (a) Overlapping peptides representing 45 VP2 N-terminal amino acids. “(K)6” denotes the addition of 6 lysine residues at the C terminus of the peptide to increase peptide solubility. (b) Peptide ELISA showing cross-reactive MAbs recognize peptides equivalent to the 15 (N15), 30 (N30), and 45 (N45) N-terminal amino acids of FMDV VP2. The 45 N-terminal amino acids of human rhinovirus VP4 (HRV-VP4) was used as a negative control; the concentration of peptides was 2 μg/ml. (c) MAb 4A3 epitope mapping (using peptides shown in panel a) identifies the cross-reactive epitope at the N terminus of VP2. (d) Reactivity of MAb 4A3 with capsid protein VP2 of all 7 serotypes in Western blotting. The 4A3 MAb produced a clearly intense band for VP2 and a weaker reaction for VP0. (e) Immunofluorescence microscopy using MAb 4A3 to detect FMDV serotype O-infected IBRS-2 cells.

Peptides representing the N-terminal 15 (VP2N15), 30 (VP2N30), or 45 (VP2N45) amino acids of FMDV VP2 were synthesized (Peptide Protein Research, United Kingdom) without modifications except for the addition of 6 lysines at the C terminus of the peptides to increase the solubility. A control peptide equivalent to a capsid sequence from the related picornavirus human rhinovirus was used (18). Eight peptides (15mer each) overlapping by 10 amino acids, covering the first 45 amino acids from the N terminus of the FMDV capsid sequence, were used for the fine mapping of the epitope (Fig. 1a).

### Serum samples.

Sera from cattle infected with FMDV O/UKG 34/2001 (19) were used to optimize the ELISA. Reference sera representing multiple strains of all serotypes from experimentally vaccinated or infected cattle were supplied by the FAO World Reference Laboratory for FMD (WRLFMD; The Pirbright Institute). The parameters of selecting serum samples were as follows. Negative (*n* = 100) samples were those collected from a negative-cohort country (during the UK 2007 outbreak). These samples were from nonvaccinated animals and proved to be negative for infection using NSP-ELISA. Positive (*n* = 72) samples were those known to be infected or vaccinated with FMDV. Selection of the positive samples was performed on the basis of their having been collected more than 7 days postvaccination or infection to ensure a positive response.

### Production of MAbs.

The following FMD viruses were used as immunogens to produce MAbs in mice and for selection of heterotypic cross-reactive MAbs as described below: serotype A Malaysia 16/97, C1 Brescia 1964, Asia 1 Nepal 29/97, A24 Cruzeiro, and O UK 31/2001.

For each immunogen, BALB/c mice were primed subcutaneously with 20 μg of purified FMD virus in Freund’s complete adjuvant and boosted intraperitoneally with the same antigen in phosphate-buffered saline (PBS) once or twice at 1-month intervals. Three days after the last boost, mice were humanely sacrificed and hybridomas were generated by fusion of splenocytes with NS0 myeloma cells following standardized procedures (20). Briefly, at least 10^8^ spleen cells were recovered from each mouse and fused with NS0 myeloma cells at a 10:1 ratio using polyethylene glycol (PEG) 4000. Fused cells diluted in Dulbecco’s modified Eagle medium, supplemented with hypoxanthine/aminopterin/thymidine and 20% fetal calf serum, were distributed over five microplates (200 μl per well). Growing colonies were observed in all wells. In order to select hybridomas secreting monoclonal antibodies specific for the immunogen, the supernatants were screened by the use of trapping ELISAs against the homologous virus strains. Selection of the intertype cross-reactive MAbs was based on results of the trapping ELISA against the homologous and heterologous virus serotypes, as previously described (21). The selected hybridoma cells were cloned by limiting dilution in order to obtain antibodies from a single cell. The supernatant from the exhausted cultures was then used as a source of MAb.

### SDS-PAGE and Western blotting.

Initial tests to verify the reactivity in Western blotting of each MAb with the homologous partially purified strain were performed as previously described (21). Later, the cross-reactivity of one representative MAb (4A3) with all FMDV serotypes was confirmed as follows.

Virus lysates from IBRS-2 cells infected with different FMDV serotypes were denatured and reduced by heating at 95°C for 5 min in Red loading buffer and dithiothreitol (DTT) (NEB). The samples were resolved through the use of 12% Tris-glycine gels and transferred to nitrocellulose membranes (GE Healthcare) (0.45 μM) using a Mini-Protean Tetra cell (Bio-Rad). Membranes were placed in blocking buffer (20 mM Tris and 150 mM NaCl [pH 7.6] with 0.1% [vol/vol] Tween 20 [TBS-T] and 1% [wt/vol] bovine serum albumin [BSA] [Melford]) for 1 h at room temperature (RT) followed by incubation with hybridoma supernatants (MAbs) and anti-mouse horseradish peroxidase (HRP)-conjugated secondary antibody (Dako) (1/5,000 in blocking buffer) in sequence for 1 h at RT. The incubations were separated by cycles of three washings with TBS-T. West Pico chemiluminescent substrate (Thermo Fisher Scientific, United Kingdom) was added to the membrane, and exposures of the membrane were collected and visualized using a G:Box Chemi XX6 system (Syngene).

### Immunofluorescence confocal microscopy.

IBRS-2 cells on 13-mm-diameter glass coverslips (VWR) were infected with FMDV type O1 Kaufbeuren (multiplicity of infection [MOI] = 2) for 3.75 h and then washed with PBS and fixed with 4% paraformaldehyde for 40 min at RT. The cells were then permeabilized for 20 min with 0.1% Triton X-100 prepared in PBS and then followed up with a 30-min blocking step using blocking buffer (Tris-buffered saline supplemented with 1 mM CaCl_2_, 0.5 mM MgCl_2_, 10% normal goat serum, and 1% fish skin gelatin). The cells were then incubated with primary antibody (mouse MAb 4A3) diluted 1/1,000 in blocking buffer for 1 h at RT. Subsequently, the cells were washed and incubated with Alexa Fluor-conjugated secondary antibody (goat anti-mouse IgG Alexa 568; Thermo Fisher Scientific, United Kingdom) in blocking buffer for 45 min at RT. After washing was performed, the cells were mounted using Vectashield mounting medium with DAPI (4,6-diamidino-2-phenylindole) (Vector Labs) and the coverslips sealed with nail varnish. All data were collected sequentially using a Leica SP8 confocal laser scanning microscope.

### Serological standard tests: virus neutralization test (VNT), liquid-phase blocking ELISA (LPBE), solid-phase competition ELISA (SPCE), and commercial kits (PrioCHECK FMDV type O, type A, and type Asia 1 antibody ELISA kits).

VNT was carried out in microplates against 100 50% tissue culture infective doses (TCID_50_) of the homologous or heterologous viruses, and results were reported as the final dilution required to neutralize 50 % of the inoculated cultures (14). The LPBE and the SPCE were carried out as described previously by Hamblin et al. (12) and by Paiba et al. (13), respectively. The cutoffs used in the VNT (log titer, 1.65), LPBE (log titer, 1.95), and SPCE (40% of inhibition) were determined according to the standard operating procedures for the WRLFMD (The Pirbright Institute, United Kingdom). PrioCHECK and SPCE ELISAs for FMDV type O, A, and Asia 1 antibody were carried out according to the instructions supplied with the kits, with 50% inhibition chosen as the cutoff.

The frequency distribution of values generated by various serological assays for the negative and the positive (vaccinated and infected animals) serum samples were plotted using GraphPad Prism (V7).

### Optimization and development of indirect VP2 ELISA.

A VP2 ELISA using peptide VP2N45 was optimized and developed using bovine reference sera. The optimal concentration of peptide and dilution of sera to be used in the test was evaluated first by checkerboard titrations using bovine sera known to be negative or strongly positive or weakly positive for antibody by existing tests. Plastic 96-well plates (Maxisorp; Nunc) were coated with 100 μl per well of the VP2N45 peptide in 0.05 M standard carbonate/bicarbonate coating buffer (pH 9.6) at 4°C overnight. Different peptide concentrations, ranging from 125 ng/ml to 4 μg/ml, were initially evaluated for test optimization. Wells were washed three times with phosphate-buffered saline (PBS) containing 0.1% Tween 20 (PBS-T) between all incubations. Wells were blocked with 200 μl blocking buffer (5% [wt/vol] skimmed milk–PBS-T) at 37°C for 1 h and incubated either with 100 μl of MAb (secreted by hybridoma cells in the supernatants, diluted 1:5) or with bovine sera (diluted 1:50 to 1 in 400 in 1% [wt/vol] skimmed milk–PBS-T) at 37°C for 1 h (Table 1). FMDV MAbs were incubated at 37°C for 1 h with 100 μl of species-specific HRP-conjugated secondary antibodies (Dako), diluted in blocking buffer (1:1,000 in the case of anti-mouse Ig conjugate or 1:15,000 for the anti-bovine-Ig conjugate). The chromogen development was mediated by the addition of 50 μl of HRP substrate (OPD [o-phenylenediamine dihydrochloride]) (Sigma FAST; Sigma, United Kingdom). The reaction was stopped after 20 min by addition of 50 μl of 1.25 M sulfuric acid, and the optical density (OD) was measured at 490 nm.

**TABLE 1:** FMDV MAbs showing cross-serotype reactivity and viral protein specificity^*a*^

MAb ID	Parent virus	Trapping ELISA							Western blotting VP target(s)
		O	A	C	ASIA1	SAT1	SAT2	SAT3	
5B2	A Malaysia 16/97	+	+	+	+	+	+	+	VP2 (+VP0)
4A3	C1	+	+	+	+	+	+	+	VP2 (±VP0)
5F10	Asia 1 Nepal 29/97	+	+	+	+	+/−	+	+	VP2 (+VP0)
4A10	A24 Cruz	+	+	+	+	+	+	+	P1
5A4	A24 Cruz	+	+	+	+	+	+	+	P1
4D1	O UK 31/01	+	+	+	+	+	+	+	VP2 (+VP0)
1D6	O UK 31/01	+	+	+	+	+	+	+	VP2 (+VP0)

a Symbols indicate positivity or negativity as follows: +, positive; −, negative; +/−, weakly positive. The data were generated from triplicate samples for each serotype. ID, identifier; VP, viral protein.

### Statistical analysis for the assay (cutoff, sensitivity, and specificity).

Sensitivity was calculated as the proportion of animals which were identified as seropositive using standard diagnostic assays following either vaccination or infection (*n* = 72) and were correctly identified as positive by the VP2 ELISA. Specificity was calculated as the proportion of samples from animals without a current or previous FMDV exposure (*n* = 100) that tested negative in the VP2 ELISA.

The sensitivity of the VP2 ELISA was compared to that of the other tests (VNT, LPBE, SPCE, and PrioCHECK) using two-proportion tests implemented in Minitab (version 18; Minitab Inc.). The assay cutoff was calculated using receiver operating characteristic (ROC) analysis as described previously by Greiner et al. (22).

## RESULTS

### Characterization of an FMDV-VP2 conserved epitope by the use of cross-reactive MAbs.

Among the multiplicity of MAbs generated from mice independently immunized with four different FMDV serotypes (A Malaysia 16/97, C1 Brescia 1964, Asia 1 Nepal 29/97, and A24 Cruzeiro or O UK 31/2001), seven MAbs were selected because of their cross-reactivity with the seven FMDV serotypes. All MAbs were characterized as nonneutralizing. Five of these MAbs strongly recognized capsid protein VP2 by Western blotting and showed a weaker reaction with VP0, while two MAbs reacted with P1 (Table 1).

Previous studies have identified the conserved N terminus of VP2 as a site for recognition by cross-reactive MAbs (15–17). We therefore tested the reactivity of the seven MAbs against peptides equivalent to the first 15 (VP2N15), 30 (VP2N30), or 45 (VP2N45) amino acids of the N terminus of VP2 from FMDV O1K (Fig. 1a). The N terminus of VP2 is known to be most highly conserved within the first 15 amino acids. The five MAbs (4D1, 1D6, 4A3, 5B2, and 5F10) identified as VP2 specific by Western blotting also reacted strongly with the VP2 peptides in ELISA (Fig. 1b). Among them, two MAbs (4A3 and 5B2) showed equivalent levels of reactivity with the three peptides, while the three remaining MAbs recognized the VP2N15 peptide with lower intensity (Fig. 1b). The MAbs were then taken forward for further characterization, in particular, for fine mapping using 15mer peptides with 10 amino acid overlaps. For simplicity, only MAb 4A3 is described in this text in more detail. As shown in Fig. 1a, MAb 4A3 reacted with the 15mer peptide that corresponded to the N terminus of VP2 and not with a 15mer starting at amino acid 6, confirming the presence of an epitope at the N terminus of VP2 (Fig. 1c). MAb 4A3 specifically detected a protein band of the expected size for VP2 in Western blotting in cell lysates from infections with all 7 serotypes (Fig. 1d), confirming that the epitope is linear, conserved, and specific for VP2. MAb 4A3 also recognized virus-infected cells when used as the primary antibody in indirect immunofluorescence microscopy of IBRS-2 cell cultures infected with type O FMDV (Fig. 1e).

### VP2N peptides detect antibodies in sera from animals infected with all serotypes of FMDV.

**FIG 2:**
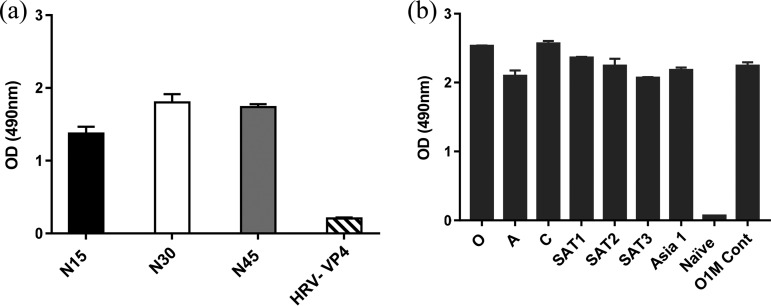
Sera from animals infected with any serotype of FMDV reacted with VP2 peptides. (a) Reactivity of serum from an animal experimentally infected with FMDV serotype O with peptides equivalent to the N-terminal 15 (N15), 30 (N30) or 45 (N45) amino acids of FMDV VP2, or the 45 N-terminal amino acids of human rhinovirus VP4 (HRV-VP4, negative control). (b) Reactivity of sera from animals vaccinated with vaccine strains of the seven serotypes with the FMDV VP2N-45 peptide.

An indirect ELISA was used with VP2N15, VP2N30, or VP2N45 peptide to assess the presence of antibodies against the N terminus of VP2 in a representative serum sample from an animal infected with type O FMDV. All three peptides captured antibodies, with the longer peptides producing a signal that was slightly higher in level (Fig. 2a). A control peptide equivalent to a capsid sequence from the related picornavirus, human rhinovirus, gave a low-level signal consistent with background.

The longer peptide VP2N45 was then used to test monovalent sera from different animals vaccinated against the seven serotypes of FMDV; the results showed that the same peptide was able to detect antibodies against all the serotypes (Fig. 2b).

### Development of a VP2 ELISA for universal detection of FMDV antibodies.

A VP2 ELISA using peptide VP2N45 was developed using reference sera. The best signal-to-noise ratio (positive/negative) was obtained using a serum dilution of 1 in 100 and a peptide concentration of 2 μg/ml (see Fig. S1 and S2 in the supplemental material). Under those optimized conditions, the cutoff for distinguishing between positive and negative signals was set at 0.4 optical density (OD) units, calculated using the average value of three independent tests and the standard negative reference serum sample used by the FAO World Reference Laboratory for FMD (WRLFMD; The Pirbright Institute) for routine FMDV diagnostics.

**FIG 3:**
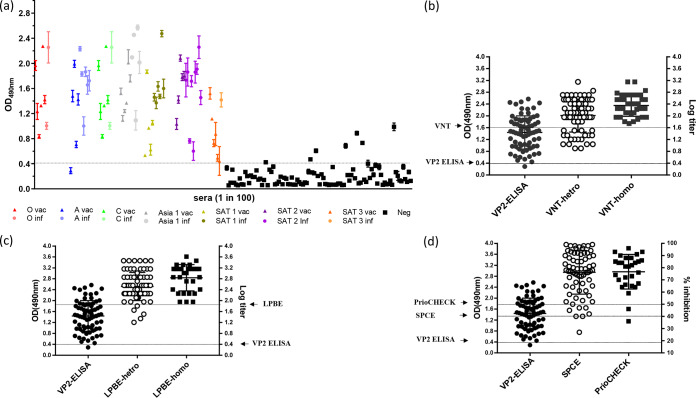
Testing reference negative and positive serum samples to determine the specificity and sensitivity of the assay. (a) Reactivity in VP2 ELISA (OD at 490 nm) of negative (Neg; black squares, *n* = 100) and positive (circles represent infections [inf], triangles represent vaccinations [vac], serotypes are represented by colors as indicated; *n* = 72) reference sera. Peptide was used at 2 μg/ml, and sera were diluted 1 in 100. (b) Distribution plots comparing results of positive sera tested with VP2 ELISA (same as data in panel a; cutoff 0.4 OD) and with homologous (*n* = 37) and heterologous (*n* = 72) VNT (cutoff = log titer 1.65). Each sample was tested in triplicate, and at least two independent experiments were performed. (c) Distribution plots comparing results of positive sera tested with VP2 ELISA (same as data in panel a; cutoff 0.4 OD) with homologous (*n* = 30) and heterologous (*n* = 72) LPBE (cutoff = log titer 1.95). (d) Distribution plots comparing results of positive sera tested with VP2 ELISA (same as data in panel a; cutoff 0.4 OD), with SPCE (*n* = 30, cutoff = 40% of inhibition) and PrioCHECK ELISA kits (*n* = 29, cutoff = 50% of inhibition).

Under the optimized assay conditions, a collection of previously characterized serum samples was tested in triplicate with two independent repeats, representing naive cattle (*n* = 100) and cattle vaccinated (*n* = 38) or infected (*n* = 34) with all seven serotypes of FMDV. The majority of vaccinated and infected (positive) samples gave a relatively strong signal (average absorbance value of 1.4), and the majority of naive (negative) samples gave a relatively weak signal (average absorbance value below 0.4) (Fig. 3a).

Seven negative samples exceeded the cutoff value of 0.4 OD units (ranging between 0.4 and 1.0 OD) and would be considered false positives, thereby producing a diagnostic specificity level of 93% for the test (95% confidence interval [CI], between 0.86 and 0.97). The signal for one positive sample (type A vaccinated) was below this cutoff and would be considered a false negative in this test, giving a sensitivity value of 99%.

### Comparison of the VP2 ELISA with existing tests (VNT, LPBE, SPCE, and PrioCHECK).

For the positive serum samples analyzed by VP2 ELISA as indicated in Fig. 3a, preexisting WRLFMD data generated using established diagnostic tests were accessed retrospectively and used to compare the performance results obtained with the VP2 ELISA. The preexisting data were generated with the following four tests: VNT to quantitate neutralizing antibodies and LPBE, SPCE, and PrioCHECK to quantitate anticapsid antibodies. The sensitivity levels of VNT, LPBE, and SPCE are dependent on close antigenic matches between the reagents used (virus/antigen and antibodies) and the serum sample being tested. Therefore, the data from VNT and LPBE were subdivided into groups and the tests were carried out with homologous reagents (same virus used to vaccinate or infect the animal) or heterologous reagents (using the same serotype but a strain different from those used to vaccinate or infect the animal). The data obtained with PrioCHECK kits were available only for samples from infections with serotypes O, A, and Asia 1.

As mentioned above, the VP2 ELISA data (Fig. 3a) contained a single false negative, equivalent to a sensitivity of 99%. In comparison, the homologous VNT data (*n* = 37) had no false negatives (sensitivity of 100%) whereas the heterologous VNT data (*n* = 72) showed a sensitivity level of 72% (Fig. 3b; see also Table 2). Similarly, the homologous LPBE data (*n* = 30) had no false negatives (sensitivity of 100%) and the heterologous LPBE data (*n* = 72) had five false negatives (sensitivity of 93%) (Fig. 3c; see also Table 2). The SPCE data (*n* = 72) had two false negatives (sensitivity of 97%) (Fig. 3d; see also Table 2), and the PrioCHECK data (*n* = 29) had two false negatives (sensitivity of 93%) (Fig. 3d; see also Table 2).

**TABLE 2:** Comparative sensitivities of VP2 ELISA and other existing serological tests

Test or sample status	Total no. of samples	No of positive samples	% sensitivity	*P* value^*a*^
VP2-ELISA	72	71	99	
VNT hetro	72	52	72	<0.0001
VNT homo	37	37	100	0.31
LPBE-hetro	72	67	93	0.092
LPBE-homo	30	30	100	0.31
SPCE-homo	72	70	97	0.55
PrioCHECK	29	27	93	0.26

Status of samples	Positive			

a Data represent results of a two-proportions test performed to compare the sensitivity of the assay to that of the VP2 ELISA.

The single false-negative sample (A Eritrea 3/98; 41 days postvaccination [dpv]) in the VP2 ELISA was also a false negative in both the heterologous VNT (log titer = 1.04) and the heterologous LPBE (log titer = 1.6) but gave a positive result in homologous VNT (log titer of 2.06) and a weakly positive result in the SPCE (52% inhibition) and PrioCHECK (65% inhibition). The sensitivity of the new test was equivalent to or better than that of PrioCHECK kits and SPCE and was significantly higher than that seen with VNT and LPBE when such assays were carried out with heterologous reagents (*P* values of <0.0001 and 0.09, respectively) using 2-proportion analysis in Minitab (Table 2).

## DISCUSSION

This report describes the development of a novel assay for the detection of antibodies against the FMDV capsid that can be used to test for seroconversion in infected or vaccinated animals. The benefits of this assay are that FMDV-specific SP antibodies from all seven serotypes can be detected without the requirement for individual specific antigen or antibody reagents such as are required for existing tests such as VNT, LPBE, and SPCE.

This assay targets a capsid epitope at the N terminus of VP2 that exhibits high sequence conservation among all seven serotypes of FMDV. Cross-reactive MAbs and overlapping peptides were used to show that the minimum sequence required for this linear epitope was VP2-N 1-DKKTE-5. This is consistent with previous studies, where structures of the FMDV capsid suggested that the N terminus of VP2 is an internal component but may be flexible, allowing it to be present at the surface to contribute to antigenicity (23–25). In addition, the production of monoclonal antibodies to VP2 N terminus in response to immunization with FMDV suggested that capsid flexibility might expose some of the internal domains of the capsid proteins to the surface, enabling them to become antigenic sites (15–17). It has also been reported previously that a purified recombinant 1AB (VP4/VP2) capsid protein was detected by antisera against all seven FMDV serotypes, indicating that the VP4/VP2 protein contained a highly conserved epitope (15). Peptides containing the VP2 N-terminal epitope were reactive with antibodies against all seven FMDV serotypes, and one (VP2N45) was selected as the basis of a novel VP2 ELISA that was evaluated with a panel of reference sera from naive (*n* = 100), vaccinated (*n* = 38), and infected (*n* = 34) cattle, representative of all the seven FMDV serotypes. Results demonstrated that the VP2 ELISA detected antibody to all serotypes with a diagnostic specificity of 93% and sensitivity of 99%. The sensitivity of the new ELISA was equivalent to or better than that of the existing tests, such as PrioCHECK kits and SPCE; sensitivity was significantly higher than that seen with LPBE and VNT carried out with heterologous reagents.

The VP2 ELISA is suitable for detection of antibodies against the capsid of FMDV either postvaccination or postinfection. The capture antigen contains a universally conserved viral epitope that is expected to be present on any isolate of FMDV; this ensures that the VP2-ELISA is able to detect FMDV antibodies regardless of the viral strain. In contrast to the biological reagents necessary in many other ELISAs, the VP2 capture antigen is a synthetic peptide, greatly facilitating standardization, continuity of supply, and reproducibility. More importantly, it does not require optimization and revalidation when serum samples from antigenically distant strains need to be tested.

Serological testing is a suitable tool for FMD surveillance. Detection of NSP antibodies currently offers the advantages of a DIVA and cross-serotype test. However, the VP2 ELISA can be used as a test that is complementary to or confirmatory for the NSP ELISA, which is especially useful in obtaining FMDV-free status after an outbreak. As with the NSP ELISA, the VP2 ELISA can also be used (i) as a front-line serosurveillance assay in areas which are normally free from FMD without vaccination, (ii) in areas which are FMD free with vaccination to conduct surveillance to achieve FMD-free status without vaccination, and (iii) at the point of import and export to confirm the absence of FMDV antibodies in animals. The test may also provide a simple approach for evaluating vaccine efficacy in experimental and field trials, although additional studies would need to be carried out to determine the cutoff that correlates to protection.

One limitation of this study might be that the potential cross-reactivity of sera positive for other, closely related picornaviruses (e.g., bovine rhinovirus 1 and 2) and of sera positive for these viruses or for other agents producing FMD-like symptoms was not tested here, and such potential cross-reactivity should be considered during validation of the test.

In conclusion, the results suggest that the VP2 ELISA developed for the detection of antibodies to FMDV has potential applications as a rapid, simple, and inexpensive test in the serodiagnosis of FMDV and in serosurveillance programs. Further validation and standardization will be needed to confirm the potential benefits of the VP2 ELISA and before using the test for diagnosis. There is a potential to expand the utility of the developed assay to other major livestock species (pigs, goats, and sheep) that are also susceptible to FMD with the development of a competitive ELISA using monoclonal antibody.

## Supplementary Material

Supplemental file 1
