# Supplementary material for: Detection of Bovine Antibodies against a Conserved Capsid Epitope as the Basis of a Novel Universal Serological Test for Foot-and-Mouth Disease
Source: J Clin Microbiol. 2020 May 26;58(6):e01527-19. doi: 10.1128/JCM.01527-19 (PMC7269384; doi:10.1128/JCM.01527-19)
Supplement: Supplemental file 1 [file JCM.01527-19-s0001.pdf]

Supplementary figures

(a)

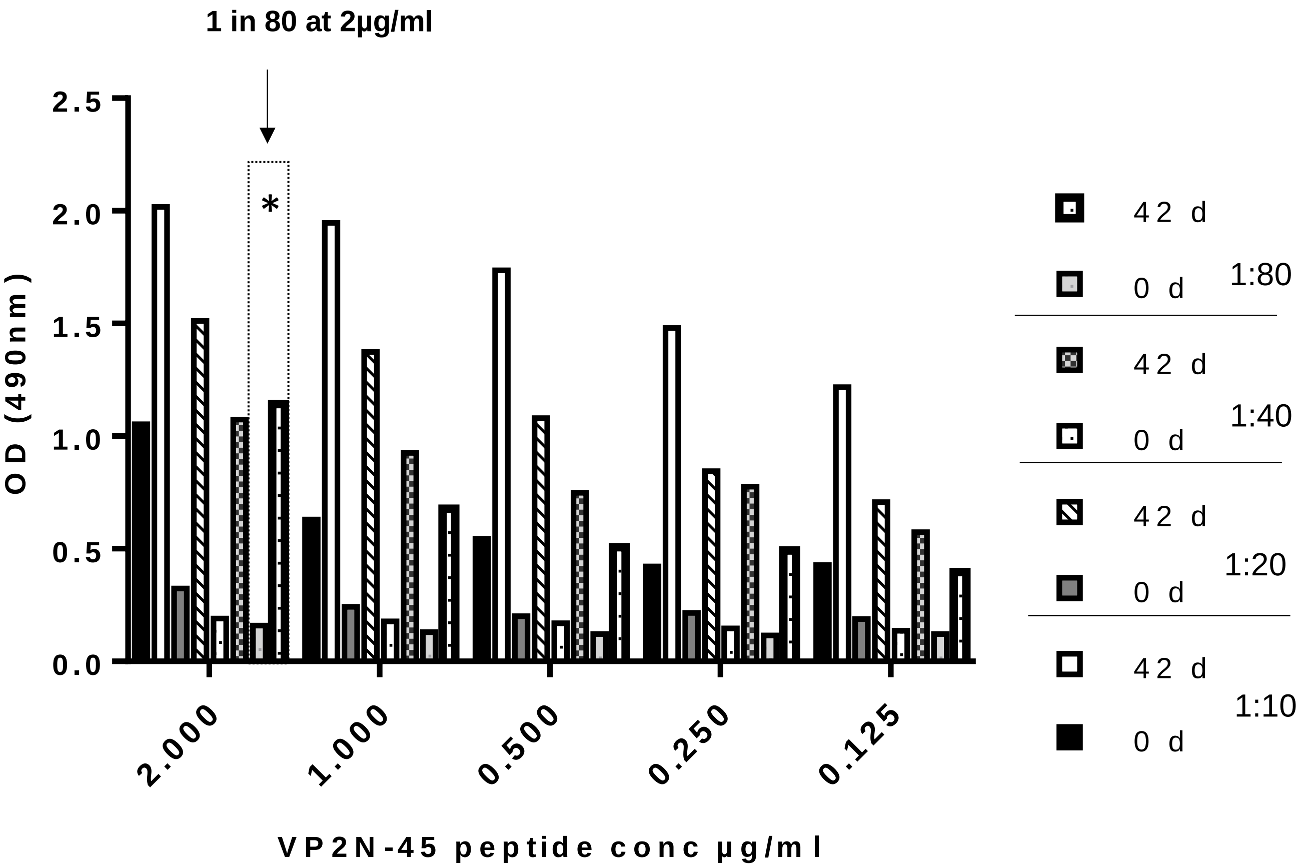

(b)

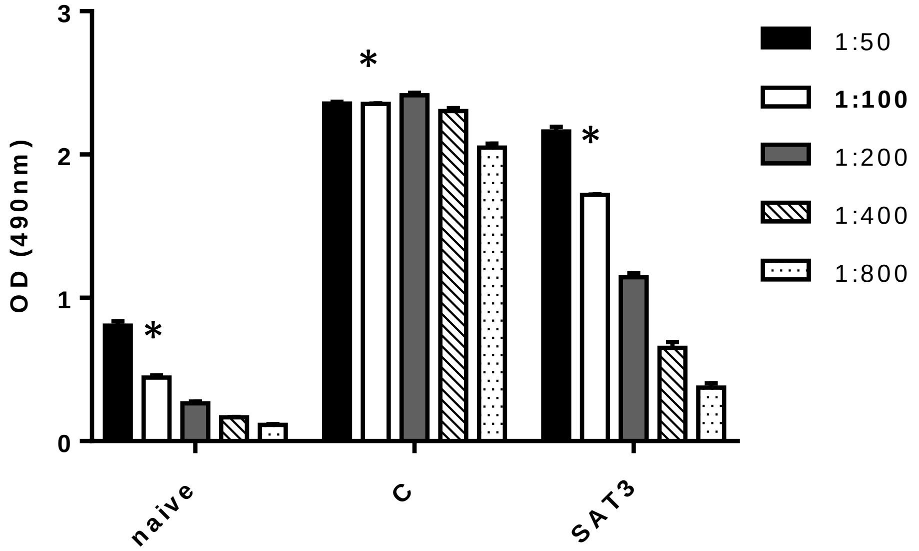

Fig S1. Optimisation of the peptide ELISA using different concentrations of peptide and dilution of the serum of serotype O from infected animal. **(a)** Checkerboard ELISA with negative (0 d) and positive (42 d) sera diluted from 1:10 to 1:80 (as shown in key) and with peptide concentration in the range 0.125-2µg/ml. The optimal conditions for signal to background are highlighted with a box (2µg/ml of peptide and 1 in 80 serum dilution). **(b)** Reactivity with VP2N45 peptide at 2µg/ml of different dilutions of a strong responder serum sample (type C) and a weak responder serum sample (type SAT3). The asterisk denotes the best conditions of peptide at 2µg/ml and sera diluted 1:100.

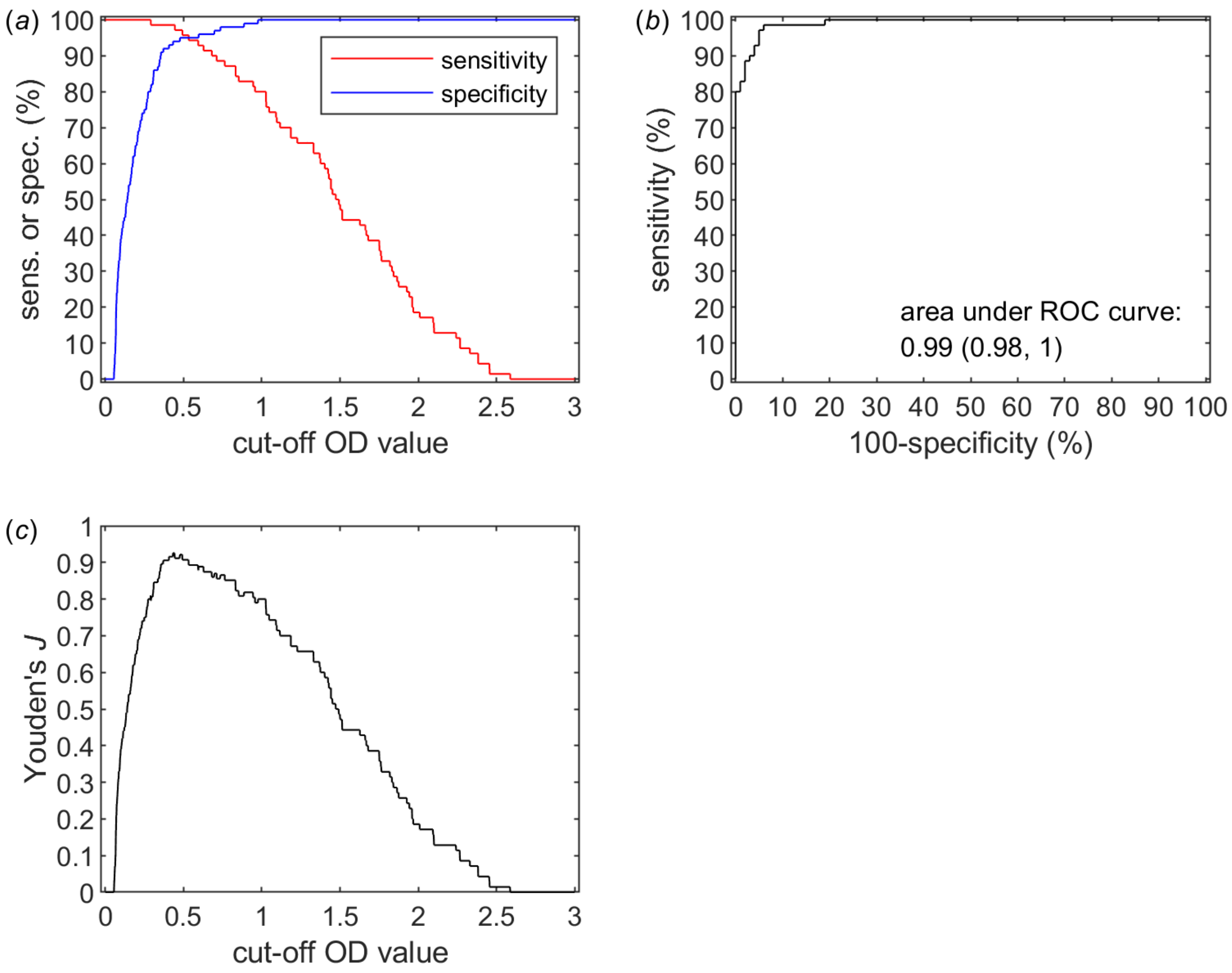

Fig S2. Receiver operating characteristic (ROC) analysis to determine the cut off value for the VP2 ELISA. (a) Sensitivity and specificity of the VP2 ELISA at different cut-offs. (b) Empirical ROC curve for the VP2 ELISA. The area under the curve is a measure of the discriminatory power of the test (with one corresponding to perfect discrimination). (c) Youden's *J* (sensitivity+specificity-1) as a function of cut-off for the VP2 ELISA. This measures the probability of making an informed decision, with the cut-off being that which maximises *J* (in this 0.43) (Greiner et al., 2000).
